# Ultrastructural characterization of GABAergic and excitatory synapses in the inferior colliculus

**DOI:** 10.3389/fnana.2014.00108

**Published:** 2014-10-15

**Authors:** Kyle T. Nakamoto, Jeffrey G. Mellott, Jeanette Killius, Megan E. Storey-Workley, Colleen S. Sowick, Brett R. Schofield

**Affiliations:** Department of Anatomy and Neurobiology, Northeast Ohio Medical UniversityRootstown, OH, USA

**Keywords:** auditory, gamma-aminobutyric acid, ultrastructure, inhibition, circuit

## Abstract

In the inferior colliculus (IC) cells integrate inhibitory input from the brainstem and excitatory input from both the brainstem and auditory cortex. In order to understand how these inputs are integrated by IC cells identification of their synaptic arrangements is required. We used electron microscopy to characterize GABAergic synapses in the dorsal cortex, central nucleus, and lateral cortex of the IC (ICd, ICc, and IClc) of guinea pigs. Throughout the IC, GABAergic synapses are characterized by pleomorphic vesicles and symmetric junctions. Comparisons of GABAergic synapses with excitatory synapses revealed differences (in some IC subdivisions) between the distributions of these synapse types onto IC cells. For excitatory cells in the IClc and ICd GABAergic synapses are biased toward the somas and large dendrites, whereas the excitatory boutons are biased toward spines and small dendrites. This arrangement could allow for strong inhibitory gating of excitatory inputs. Such differences in synaptic distributions were not observed in the ICc, where the two classes of bouton have similar distributions along the dendrites of excitatory cells. Interactions between excitatory and GABAergic inputs on the dendrites of excitatory ICc cells may be more restricted (i.e., reflecting local dendritic processing) than in the other IC subdivisions. Comparisons across IC subdivisions revealed evidence for two classes of GABAergic boutons, a small GABAergic (SG) class that is present throughout the IC and a large GABAergic (LG) class that is almost completely restricted to the ICc. In the ICc, LG, and SG boutons differ in their targets. SG boutons contact excitatory dendritic shafts most often, but also contact excitatory spines and somas (excitatory and GABAergic). LG synapses make comparatively fewer contacts on excitatory shafts, and make comparatively more contacts on excitatory spines and on somas (excitatory and GABAergic). LG boutons likely have a lemniscal origin.

## INTRODUCTION

The IC is involved in a wide range of auditory functions. The majority of ascending auditory information converges in the IC, so it is associated with most aspects of auditory perception. The IC is also involved in orienting behavior and acoustic guidance of movement, reflecting its connections with motor centers such as the SC and Pn (Huffman and Henson, 1990). The responses of neurons in the IC are shaped by a convergence of excitatory and inhibitory inputs (Sivaramakrishnan and Oliver, 2001; Kelly and Caspary, 2005; Oliver, 2005; Saldaña and Merchán, 2005; Schofield, 2005; Wu, 2005; Cant and Benson, 2006; Pollak et al., 2011). Glutamate is the major excitatory neurotransmitter, whereas GABA and glycine are the major inhibitory neurotransmitters. All three neurotransmitters are associated with ascending auditory inputs to the IC. In addition, the IC contains glutamatergic and GABAergic cells that, through local and commissural axons, also contribute to IC circuitry (Saldaña and Merchán, 2005; Chandrasekaran et al., 2013; Grimsley et al., 2013). Descending projections from auditory cortex are themselves glutamatergic, but they contact both glutamatergic and GABAergic IC cells, and thus initiate both excitatory and inhibitory effects in the IC (Mitani et al., 1983; Syka and Popelár, 1984; Torterolo et al., 1998; Yan and Ehret, 2001; Suga, 2008; Nakamoto et al., 2010; Anderson and Malmierca, 2013). Relatively little is known about the distribution of excitatory and inhibitory inputs on the dendrites and somas of IC cells. Characterizing these distributions is fundamental to understanding how IC cells integrate information and perform the multitude of functions attributed to the IC.

This report builds on previous descriptions of excitatory synapses in the IC (Nakamoto et al., 2013a,b). In those studies, we distinguished three different types of excitatory synapses based on differences in presynaptic bouton morphology and distribution among IC subdivisions. The excitatory boutons, which together constitute about 60% of the synapses in the IC, formed synapses with somas, dendritic shafts, and spines on cells in all IC subdivisions. Significantly, many of the excitatory synapses were located in close proximity to inhibitory synapses, presumably reflecting the many physiological demonstrations of interactions between excitatory and inhibitory inputs in IC cells (reviewed by Pollak et al., 2011). The present report focuses on the GABAergic synapses and their distribution relative to the excitatory synapses on IC cells.

## EXPERIMENTAL PROCEDURES

Experiments were performed on six adult pigmented guinea pigs of either gender weighing 400–900 g (Elm Hill Breeding Laboratories, Chelmsford, MA, USA). All procedures were approved by the Institutional Animal Care and Use Committee (IACUC) and administered following the National Institutes of Health guidelines for the care and use of laboratory animals. In accordance with these guidelines, all efforts were made to minimize the number of animals used and their suffering. Five of the six animals used for this study underwent survival surgery for injection of anatomical tracers into the auditory cortex prior to perfusion. The procedures and results for the tracer injections are described in a separate report (Nakamoto et al., 2013b) which analyzed excitatory synapses in the IC. The use of tissue from those animals allowed us to minimize the number of animals needed for the present study.

### PERFUSION AND SECTIONING

Each animal was sacrificed with an overdose of sodium pentobarbital (440 mg/kg; i.p., Euthasol, Virbac Inc., Fort Worth, TX, USA) or isoflurane (inhalation until cessation of breathing; Aerrane, Baxter, Deerfield, IL, USA). The animal was perfused through the aorta with Tyrode’s solution, followed by 2% paraformaldehyde and 2% glutaraldehyde in 0.1M PB (pH 7.4). The brain was then removed and stored overnight at 4∘C in 2% paraformaldehyde and 2% glutaraldehyde in PB. The following day, tissue blocks containing the IC were cut into 50 μm parasagittal sections with a Vibratome. Sections were collected in six series and processed as described below or placed in freezing buffer and stored for future processing.

### IDENTIFICATION OF IC SUBDIVISIONS

A detailed description of the methods used for identification of IC subdivisions has been previously reported (Nakamoto et al., 2013a). Briefly, one series of sections from each animal was stained for NADPH-d activity (Dawson et al., 1991). The stained sections were mounted on slides, dried overnight, and then coverslipped with DPX (Aldrich Chemical Company, Inc., Milwaukee, WI, USA). The NADPH-d stain reflects the distribution of neuronal nitric oxide synthase and can be used to distinguish IC subdivisions in guinea pigs (Coote and Rees, 2008).

### PROCESSING FOR ELECTRON MICROSCOPY

**FIGURE 1:**
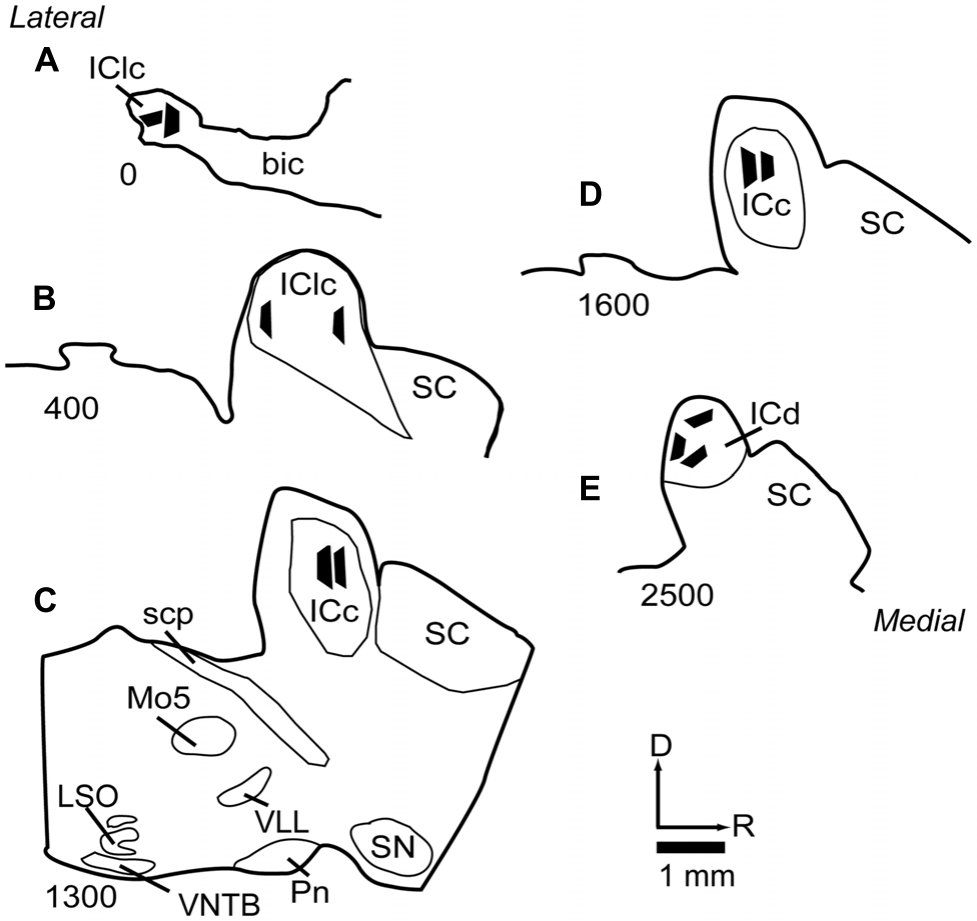
**Line drawings of parasagittal sections through the IC showing the locations of the 11 tissue blocks collected for analysis.** Several blocks were collected from each of three subdivisions of the IC. Each black trapezoid represents the position of a single tissue block. Samples were taken from multiple sections across several animals, but are drawn on a single representative series of sections for ease of comparison. **(A–E)** Sections are arranged from lateral to medial; the numbers at lower left of each section indicate approximate distance of the section, in μm, from the lateral-most section in the series. See list for abbreviations.

Selected sections were post-fixed in 2% osmium tetroxide, embedded in Durcupan resin (Electron Microscopy Sciences, Fort Washington, PA, USA) and flat-mounted between sheets of Aclar Embedding Film (Ted Pella, Inc., Redding, CA, USA). The sections were then examined in a light microscope and compared to NADPH-d stained sections in order to determine the boundaries of the IC subdivisions. An area up to 1 mm on a side and located completely within a particular IC subdivision was trimmed from the section with a scalpel and glued onto a resin base with cyanoacrylate (Krazy Glue, Columbus, OH, USA). The positions of the tissue blocks were plotted and superimposed on a series of NADPH-d stained sections (**Figure 1**), using a Zeiss Axioplan 2 microscope with a Neurolucida system (MBF Bioscience, Williston, VT, USA). For samples from the ICc, we selected regions with low NADPH-d staining, well within the borders of the ICc. The locations of the ICc samples are shown in (**Figures 1C,D**). For analysis of the IClc, we took samples from regions associated with strong NADPH-d staining and located in very lateral sections of the IC (**Figures 1A,B**). Finally, for analysis of dorsal cortex, we took samples from areas associated with strong NADPH-d staining and located in the dorsomedial part of the IC (**Figure 1E**).

Ultrathin sections (∼100 nm, gold–silver interference color) were cut from each tissue block using an ultramicrotome (UC6 Ultramicrotome, Leica Microsystems, Buffalo Grove, IL, USA) and every seventh section was collected. Based upon our measurements of the maximum size of the synaptic densities in the guinea pig IC we determined that a spacing of 600 nm was sufficient to avoid taking multiple sections of the same synapse. Each section was collected on a 300-mesh nickel grid and immunostained for GABA (Coomes et al., 2002; Nakamoto et al., 2013a). Briefly, grids with ultrathin sections were incubated overnight in anti-GABA antibody (rabbit anti-GABA, Sigma, St. Louis, MO, USA), rinsed, placed into a secondary antibody conjugated to 15 nm gold particles (goat anti-rabbit, Ted Pella, Inc., Redding, CA, USA) and finally stained with uranyl acetate and, in some cases, Reynolds’s lead citrate (Reynolds, 1963).

### ELECTRON MICROSCOPY, IMAGE PREPARATION, AND IMAGE ANALYSIS

Ultrastructure was observed with a transmission electron microscope (JEM-100S; JEOL, Peabody, MA, USA) at 60 kV and 15,000–40,000 magnification. Images were recorded on Kodak SO-163 film (Kodak, Rochester, NY, USA). The negatives were then scanned at a resolution of 1200–2000 pixels/inch using a large-format backlit scanner (ScanMaker i800, Microtek, Santa Fe Springs, CA, USA) to produce digital images for analysis. Adobe Photoshop and Adobe Illustrator (Adobe, San Jose, CA, USA) were used to adjust brightness and contrast levels, to add colors to facilitate descriptions, and to arrange and label photographs. The images were analyzed with ImageJ (Abramoff et al., 2004) for measurements of profile area. The minimum diameter of the profiles was used in the analyses. Line graphs were generated with Excel and schematics were generated with PowerPoint (Microsoft Corporation, Redmond, WA, USA).

Each ultrathin tissue section was examined methodically at 20 000 magnification by starting in one corner of the section and moving back and forth across the section in order to photograph every potential GABAergic synapse. Images were collected in multiple sessions until the samples exceeded 100 GABAergic synapses from each IC subdivision. Overall, 18 sections were examined in order to collect the necessary number of GABAergic synapses. The final sample totaled 373 synapses (116 from ICc; 114 from IClc; 143 from ICd).

### IDENTIFICATION OF GABA IMMUNOREACTIVITY

**FIGURE 2:**
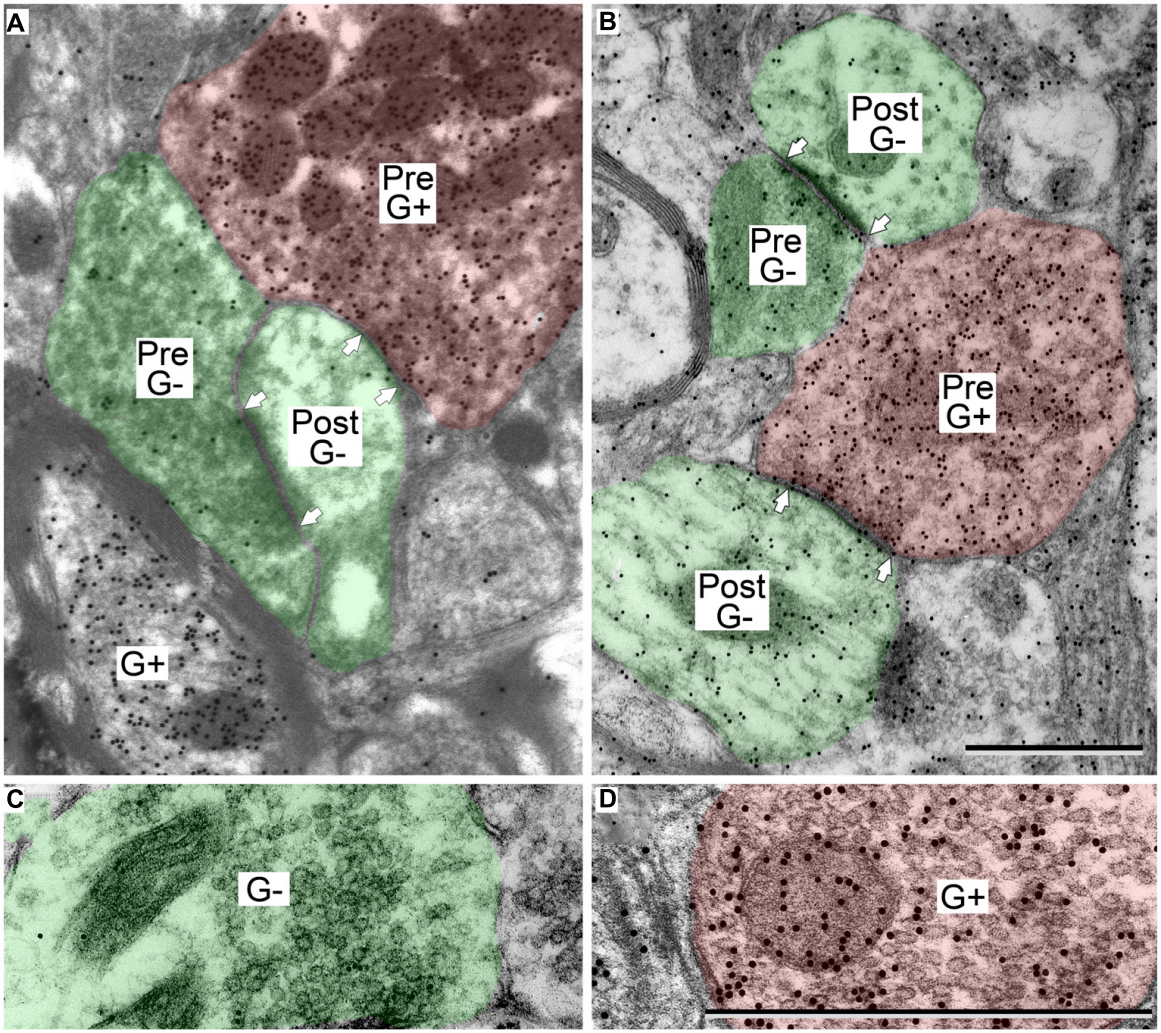
**Electron micrographs showing examples of GABA-positive and GABA-negative synapses in the IC.** Presynaptic (Pre) and postsynaptic (Post) profiles are labeled and synaptic densities are bracketed by arrows placed in the postsynaptic profiles. GABA immunoreactivity is demonstrated by the increased density of gold particles (black dots, G+) relative to surrounding tissue. **(A)** Image of a GABA-negative (G-) synapse (green; Pre) and a GABA-positive synapse (red; Pre) contacting the same GABA-negative dendritic spine (green; Post). A GABA-positive myelinated axon is visible at lower left (G+). **(B)** Image of a GABA-negative synapse and a GABA-positive synapse (note arrows). Both postsynaptic profiles are GABA-negative dendritic shafts (green; Post). The GABA-positive synapse has a GABA-positive presynaptic profile (red; Pre), and a symmetric synaptic density. The GABA-negative synapse has a GABA-negative presynaptic profile (green; Pre) and an asymmetric synaptic density. **(C,D)** Higher magnification images to show the round vesicles in an excitatory presynaptic bouton **(C)** and the pleomorphic vesicles in a GABA-positive presynaptic bouton **(D)**. Coloring and symbols are the same throughout the subsequent micrographs. Scale bars = 1 μm.

GABA-positive profiles (**Figure 2**, G+) were identified by visual comparison of the number of gold particles over a profile relative to background labeling in surrounding tissue (Coomes et al., 2002; Nakamoto et al., 2013a,b). As with many immunostaining procedures, background staining can vary across sections and across cases, so all assessments were done by comparison with nearby regions. With this approach, immunopositive and immunonegative profiles were easily distinguished. The technical considerations of the GABA immunoreactivity have been discussed in detail in previous reports (Nakamoto et al., 2013a,b).

## RESULTS

We first describe the general morphology of GABAergic synapses in the IC. We then provide details about the GABAergic synapses in the three major subdivisions of the IC. Finally we compare the targets of GABAergic and excitatory synapses in each of the subdivisions.

### GENERAL MORPHOLOGY OF GABAergic SYNAPSES IN THE IC

GABAergic synapses were identified by presynaptic profiles that were GABA-positive and contained pleomorphic vesicles, and postsynaptic densities that are less prominent than those in excitatory synapses (i.e., a symmetric synapse; **Figure 2**, synapses with red presynaptic profiles). In contrast, excitatory synapses have GABA-negative presynaptic boutons that contain round synaptic vesicles and have thicker postsynaptic densities (**Figure 2**, synapses with green presynaptic profiles; Peters et al., 1991; Nakamoto et al., 2013a). A third group of boutons contained pleomorphic vesicles and formed symmetric junctions, but were GABA-negative. This morphology is consistent with inhibitory transmission. These synapse were likely glycinergic and were not included in our data collection procedures. For the rest of the article GABA-positive profiles will be referred to as GABAergic and will be colored red in the figures. The postsynaptic profiles were either GABA-positive or GABA-negative. We interpret the GABA-positive postsynaptic profiles as belonging to GABAergic IC cells. The GABA-negative postsynaptic profiles are likely to be excitatory, as there appear to be only glutamatergic and GABAergic cells in the IC (Kelly and Caspary, 2005; Ito et al., 2011). However, presumptive excitatory synapses (i.e., GABA-negative boutons with round vesicles and asymmetric synaptic junctions) are likely to include glutamatergic synapses (almost certainly the majority) but may also include non-glutamatergic synapses (perhaps representing cholinergic or serotoninergic inputs). We have previously referred to this population as “excitatory” synapses (Nakamoto et al., 2013a). For the rest of the article, presumptive excitatory profiles (GABA-negative postsynaptic profiles and excitatory presynaptic boutons) will be colored green in the figures.

**FIGURE 3:**
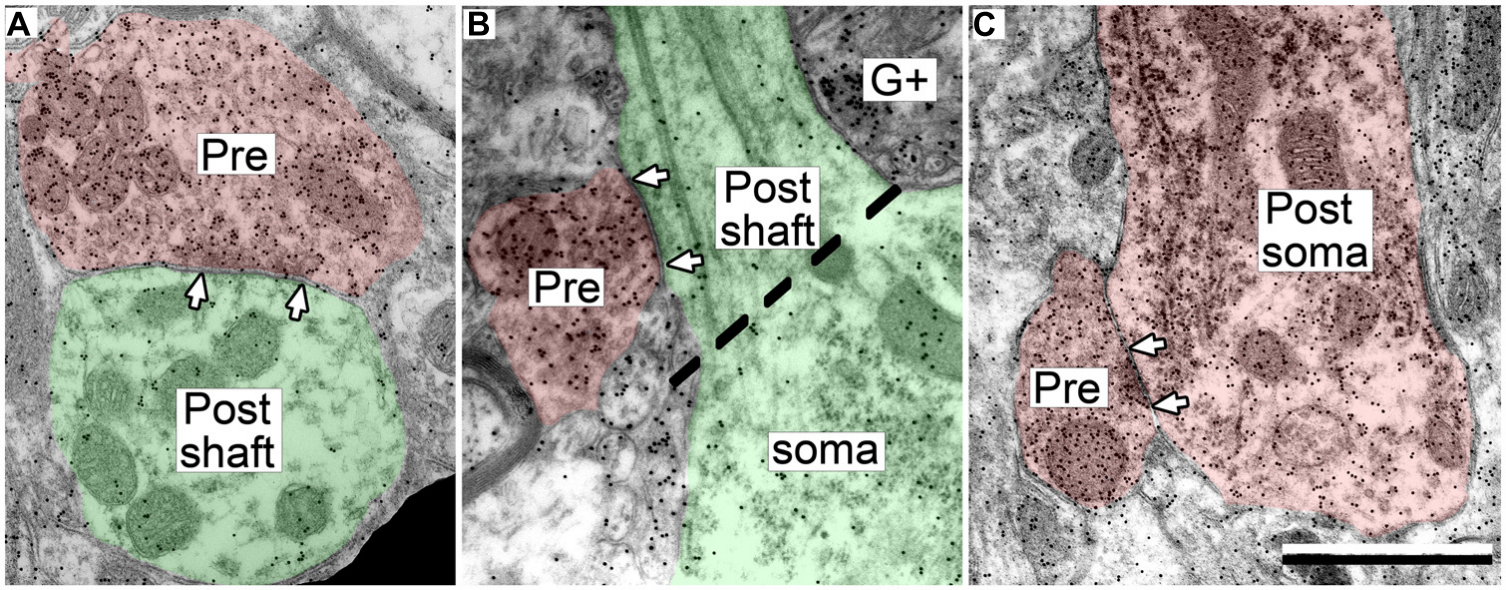
**Electron micrographs showing examples of GABAergic synapses with various postsynaptic profiles. (A)** A GABAergic bouton that formed a synapse with an excitatory dendritic shaft in the ICc. **(B)** A GABAergic bouton that formed a synapse with an excitatory dendritic shaft in the ICd. The synapse formed at the base of the dendrite, just as it arose from the soma (separated by the dashed line). **(C)** A GABAergic bouton that formed a synapse with a GABA-positive soma in the ICd. Scale bar = 1 μm. Conventions as in **Figure 2**.

The postsynaptic profiles of GABAergic synapses included GABAergic and excitatory targets, which were identified as spines, dendritic shafts, and somas. Spines (Post, **Figure 2A**) can be distinguished from dendritic shafts (Post, **Figure 2B**) by the lack of microtubules, the presence of electron dense “fluffy material” and, in some instances, the presence of thin-walled sacs (Rockel and Jones, 1973; Peters et al., 1991; Nakamoto et al., 2013a) in the spines. Somas can be distinguished from shafts by the lack of uniformly oriented organelles and in some sections by the presence of a nucleus (**Figure 3**, compare the organelles in the soma and the shaft; Peters et al., 1991). For the GABAergic synapses, the majority of postsynaptic profiles were excitatory (**Figures 3A,B**), though contacts onto GABAergic profiles (**Figure 3C**) also occurred.

While analyzing the GABAergic synapses we noticed that they often occurred on proximal dendritic shafts, adjacent to the soma (e.g., **Figure 3B**). Consistent with this observation, many of the postsynaptic shafts appeared relatively large, particularly in comparison to the postsynaptic shafts of excitatory synapses in the IC. Such a difference implies differential distribution of the synapse types on dendritic shafts that taper in diameter with distance from the soma. In order to investigate this possibility, we measured the minimum diameter of the postsynaptic dendritic profiles for the GABAergic synapses in each subdivision. For comparison, we also analyzed the diameters of the dendritic postsynaptic profiles of excitatory synapses collected in our previous study (Nakamoto et al., 2013a). The results are described separately for each IC subdivision.

### GABAergic SYNAPSES IN THE LATERAL CORTEX OF THE IC

**FIGURE 4:**
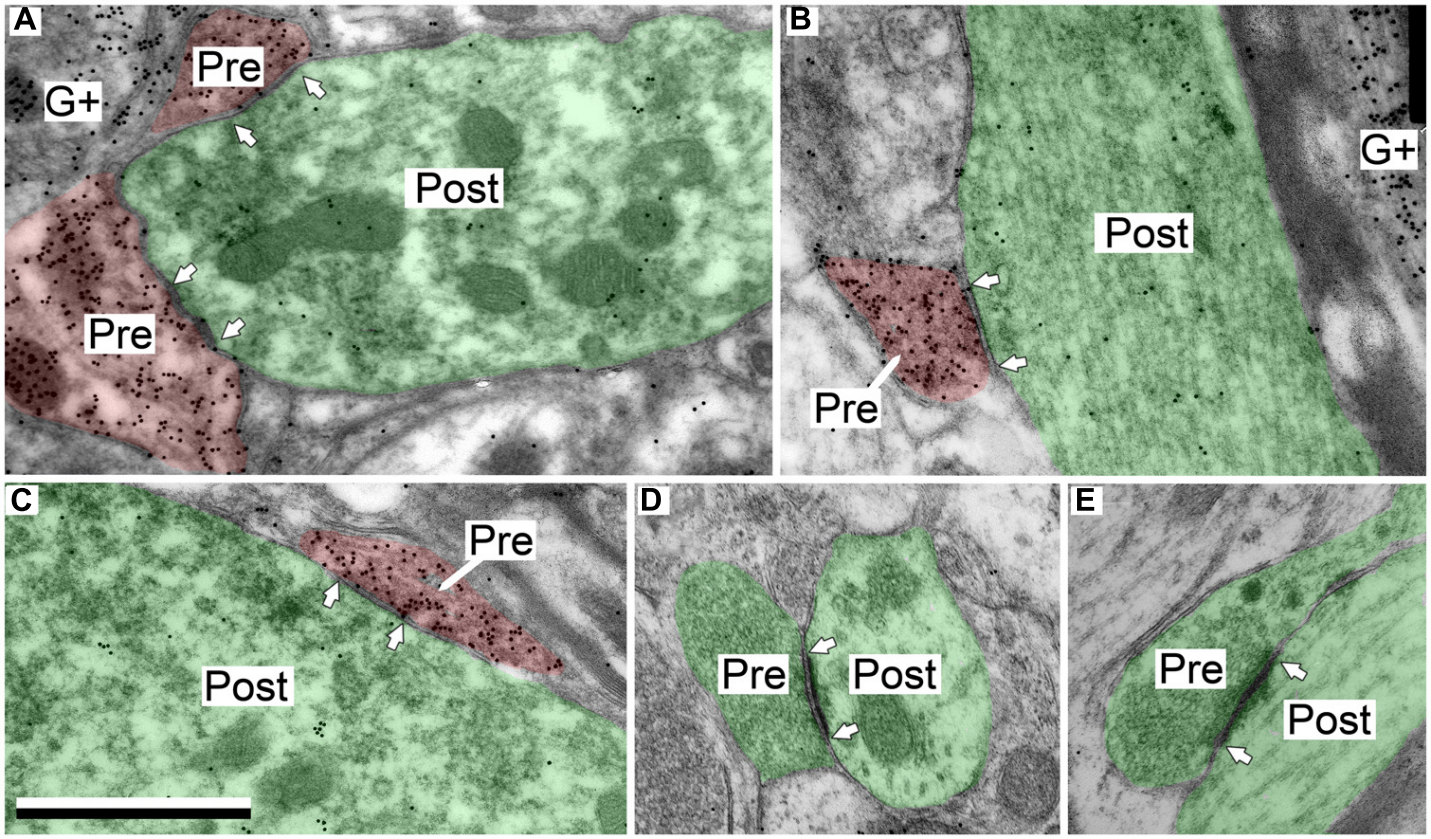
**Electron micrographs showing examples of GABAergic and excitatory synapses in the IClc. (A,B)** GABAergic boutons contacting large excitatory dendritic shafts. **(C)** GABAergic bouton contacting an excitatory soma. **(D,E)** Excitatory boutons contacting small excitatory dendritic shafts. Scale bar = 1 μm. Conventions as in **Figure 2**.

In the IClc the GABAergic presynaptic boutons typically were small (<0.9 μm^2^ in area; **Figures 4A–C**). These boutons formed synapses with both excitatory and GABAergic profiles, though the majority (85%) were excitatory. Most of these postsynaptic profiles were dendritic shafts, but contacts also occurred with spines and somas (**Table 1**, IClc). A small portion (15%) of the GABAergic synapses in IClc had GABAergic postsynaptic profiles. These postsynaptic profiles included shafts and somas (**Table 1**, IClc). Our sample did not include any GABAergic synapses with GABAergic spines as the postsynaptic profile, though we did observe GABAergic spines in the neuropil.

**Table 1:** Distributions of target profile types for GABAergic boutons and excitatory boutons in each of three IC subdivisions.

		Excitatory targets				GABAergic targets				
		Soma (%)	Shaft (%)	Spine (%)	Exc. subtotal (%)	Soma (%)	Shaft (%)	Spine (%)	GABA subtotal (%)	Total
IClc	GABAergic boutons	8	67	10	**85**	5	10	0	**15**	100
	Excitatory boutons	0	59	32	**91**	1	8	0	**9**	100
ICd	GABAergic boutons	5	69	17	**91**	3	5	1	**9**	100
	Excitatory boutons	1	41	50	**92**	1	4	3	**8**	100
ICc	GABAergic boutons	10	56	21	**87**	9	4	0	**13**	100
	Excitatory boutons	1	62	16	**79**	5	15	1	**21**	100

*Each row totals 100%. Exc., excitatory; ICc, central nucleus of the IC; ICd, dorsal cortex of the IC; IClc, lateral cortex of the IC.*

### GABAergic AND EXCITATORY SYNAPSES HAVE DIFFERENT DISTRIBUTIONS ON IClc CELLS

Comparison of these data with our previous data on excitatory synapses suggested that GABAergic and excitatory boutons are distributed differently with respect to postsynaptic profile identity (i.e., spine, dendritic shaft, soma) and with respect to GABAergic or excitatory targets. Furthermore it appeared that contacts onto dendritic shafts might vary according to the diameter of the shaft. In order to assess this possibility, we measured the diameter of the postsynaptic shafts for both the GABAergic synapses and the excitatory synapses in the IClc.

**FIGURE 5:**
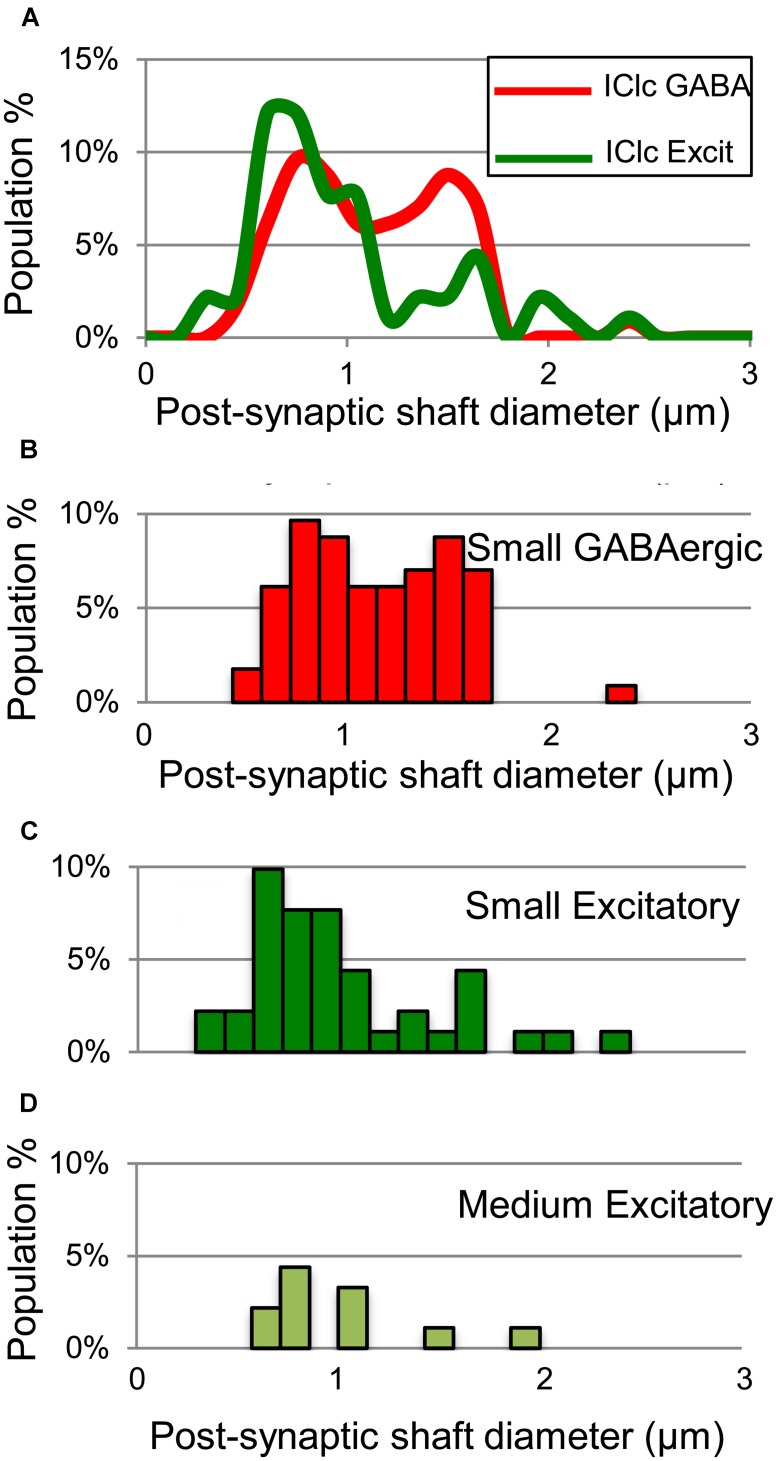
**Plots showing the diameters of excitatory dendritic shafts that are part of GABAergic synapses (red) or excitatory synapses (green) in the IClc. (A)** Line graphs comparing the diameters of target dendrites for all GABAergic synapses (red) vs. all excitatory synapses (green). **(B)** Histograms showing the diameters of the post-synaptic dendritic shafts for the GABAergic synapses. The y-axis is the population percentage for all GABAergic synapses in the IClc. **(C,D)** Histograms showing the diameters of the post-synaptic dendritic shafts for the Small or Medium subtypes of excitatory synapses. The y-axis is the population percentage for all excitatory synapses in the IClc. Excit – excitatory.

Both GABAergic and excitatory synapses form with excitatory targets much more often than with GABAergic targets (**Table 1**, IClc). The remaining points are best considered separately for the excitatory target cells and the GABAergic target cells. The GABAergic and excitatory synapses on excitatory targets are distributed differently (**Table 1**, IClc, excitatory targets). A larger percentage of excitatory boutons contact spines (32% vs. 10%). In addition, both GABAergic and excitatory synapses occur most often on shafts (67% vs. 59%), but GABAergic synapses form on shafts that, on average, are significantly larger than those contacted by excitatory synapses [Welch’s *t*-test; *t*(123) = 2.166, *p* = 0.032, **Figure 5A**]. This difference in target diameter was also apparent when we compared GABAergic synapses to the subclasses of excitatory synapses (Nakamoto et al., 2013a). Briefly, excitatory boutons in the IClc can be classified as small (SE) or medium (ME) based on profile size and mitochondrial content. GABAergic synapses frequently formed on shafts of a wide range of sizes (**Figure 5B**), while SE (**Figure 5C**) and ME (**Figure 5D**) boutons formed synapses primarily on smaller shafts.

Differences between GABAergic contacts and excitatory contacts onto GABAergic neurons were less dramatic than those onto the excitatory cells (**Table 1**, IClc, GABAergic targets). The apparent paucity of synapses onto the GABAergic targets reflects the frequency with which each bouton class contacts GABAergic vs. excitatory cells, uncorrected for the number of such cells in the IC (i.e., excitatory cells are approximately three times more numerous than GABAergic cells in the IC; Oliver et al., 1994; Merchán et al., 2005; Mellott et al., 2014). Both GABAergic and excitatory boutons contacted dendritic shafts and somas of GABAergic cells, but the GABAergic boutons are more strongly biased toward the somas.

### GABAergic SYNAPSES IN THE DORSAL CORTEX OF THE IC

**FIGURE 6:**
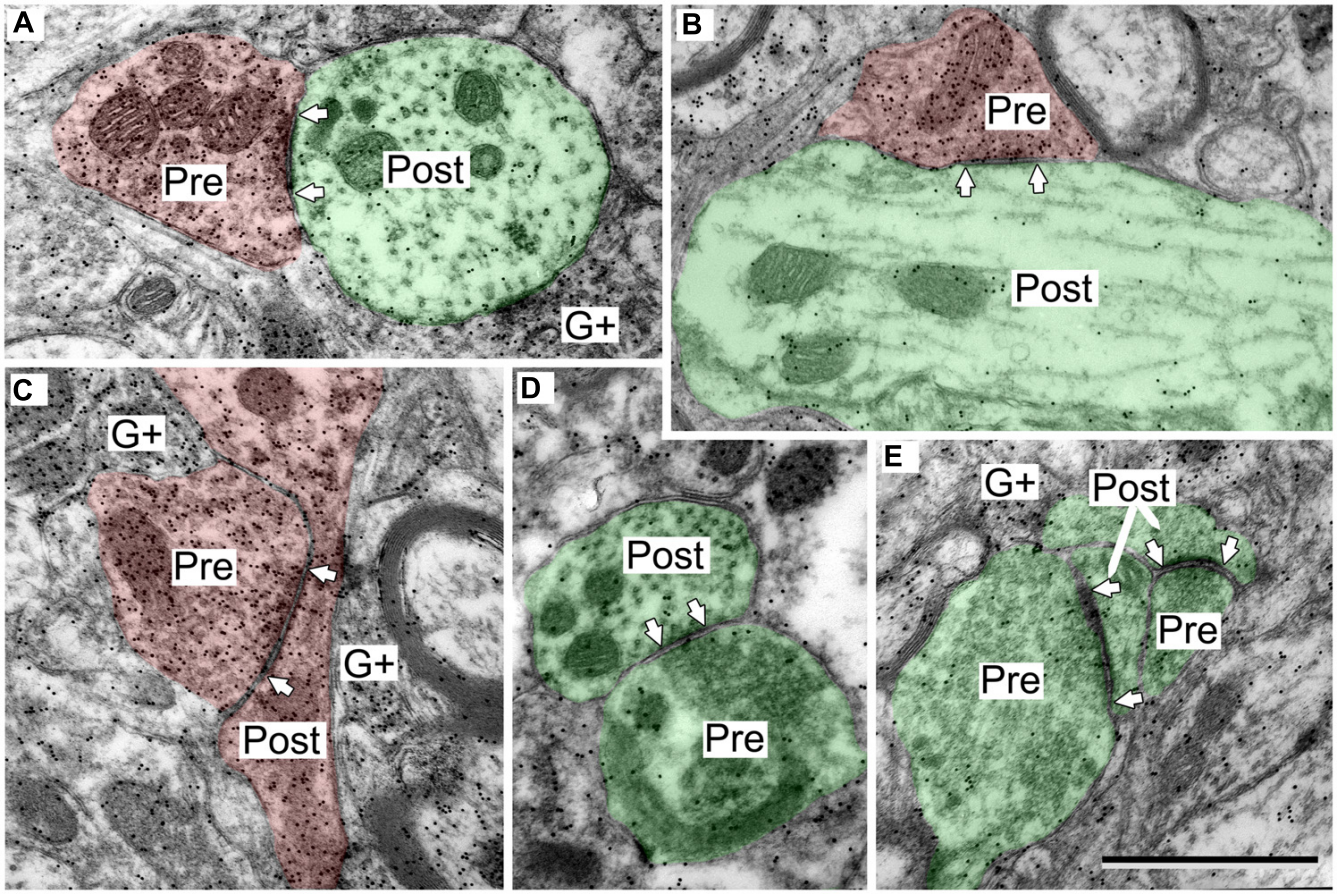
**Electron micrographs showing examples of GABAergic and excitatory synapses in the ICd. (A,B)** GABAergic boutons contacting excitatory shafts. **(C)** GABAergic bouton contacting a GABAergic spine that branches from a shaft. **(D)** Excitatory bouton contacting an excitatory shaft. **(E)** Two excitatory boutons contacting excitatory spines. Scale bar = 1 μm. Conventions as in **Figure 2**.

In the ICd the GABAergic presynaptic boutons typically were small (<0.9 μm^2^ in area; **Figures 6A–C**). These boutons formed synapses with both excitatory and GABAergic profiles (**Table 1**, ICd). Similar to the IClc, the majority (91%) of the postsynaptic targets were excitatory. Most of these postsynaptic profiles were dendritic shafts, but contacts also occurred with spines and somas. A small portion (9%) of the GABAergic synapses in ICd had GABAergic postsynaptic profiles. These postsynaptic profiles included shafts, somas, and spines (**Figure 6C**).

### GABAergic AND EXCITATORY SYNAPSES HAVE DIFFERENT DISTRIBUTIONS ON ICd CELLS

In the ICd, comparisons of GABAergic synapses with our previous data on excitatory synapses also suggested that these boutons are distributed differently with respect to postsynaptic profile identity, including the size of contacted dendritic shafts. We compared these distributions in the same manner as we did in the IClc. As in IClc, both GABAergic and excitatory synapses form with excitatory targets much more often than with GABAergic targets (**Table 1**, ICd).

**FIGURE 7:**
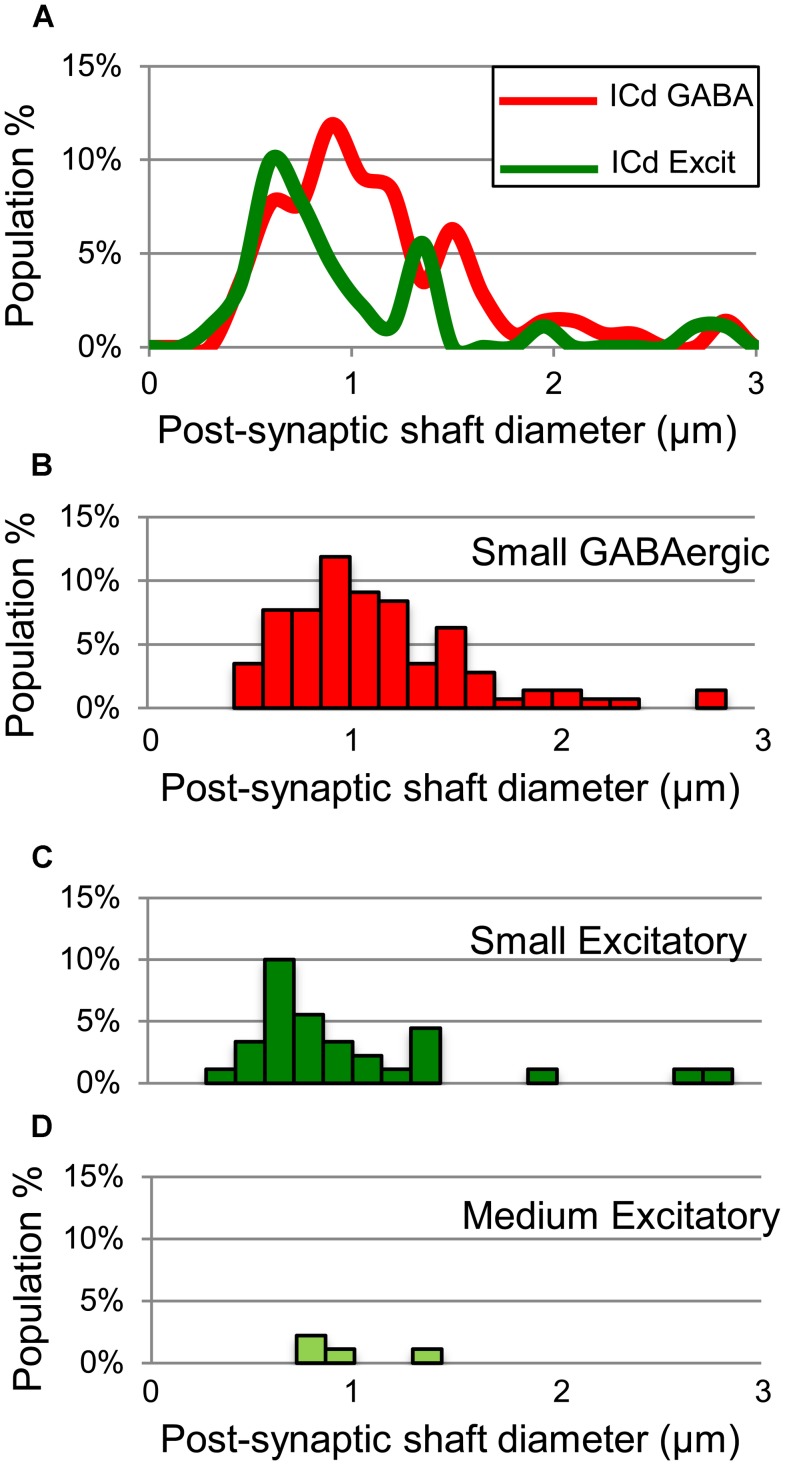
**Plots showing the diameters of excitatory dendritic shafts that are part of GABAergic synapses (red) or excitatory synapses (green) in the ICd. A)** Line graphs comparing the diameters of target dendrites for all GABAergic synapses (red) vs. all excitatory synapses (green). **(B)** Histograms showing the diameters of the post-synaptic dendritic shafts for the GABAergic synapses. The y-axis is the population percentage for all GABAergic synapses in the ICd. **(C,D)** Histograms showing the diameters of the post-synaptic dendritic shafts for the Small or Medium subtypes of excitatory synapses. The y-axis is the population percentage for all excitatory synapses in the ICd. Excit – excitatory.

GABAergic and excitatory synapses on excitatory targets are distributed differently (**Table 1**, ICd, excitatory targets). Half the excitatory boutons contact spines, while GABAergic boutons contact spines less frequently (50% vs. 17%). In contrast, the majority of GABAergic synapses occur on dendritic shafts, while less than half of the excitatory synapses occur on dendritic shafts (69% vs. 41%). In addition, the GABAergic synapses form on significantly larger excitatory dendritic shafts than do excitatory synapses [Welch’s *t*-test; *t*(124) = 2.166, *p* = 0.031, **Figure 7A**; compare the diameter of the shafts, perpendicular to the microtubules, in **Figures 6A,B** vs. **Figure 6D**]. This difference in target diameter was also apparent when we compared GABAergic synapses to the subclasses of excitatory synapses (Nakamoto et al., 2013a). GABAergic synapses frequently formed on shafts of a wide range of sizes (**Figure 7B**), while SE (**Figure 7C**) and ME (**Figure 7D**) boutons formed synapses primarily on smaller shafts. Overall GABAergic synapses form more frequently on shafts, including larger shafts, and excitatory synapses form more frequently on spines and smaller shafts.

In the ICd stark differences in the distributions of GABAergic and excitatory contacts onto GABAergic neurons were not observed (**Table 1**, ICd, GABAergic Targets). As in IClc, excitatory cells are approximately three times more numerous than GABAergic cells in the ICd. We do not claim that there are no differences in these contacts onto GABAergic neurons, but rather we did not find any in our small sample of contacts on GABAergic cells. It can conclud that both GABAergic and excitatory boutons contacted the shafts, somas and spines of GABAergic targets.

### THE CENTRAL NUCLEUS OF THE IC CONTAINS BOTH LARGE AND SMALL GABAergic BOUTONS

**FIGURE 8:**
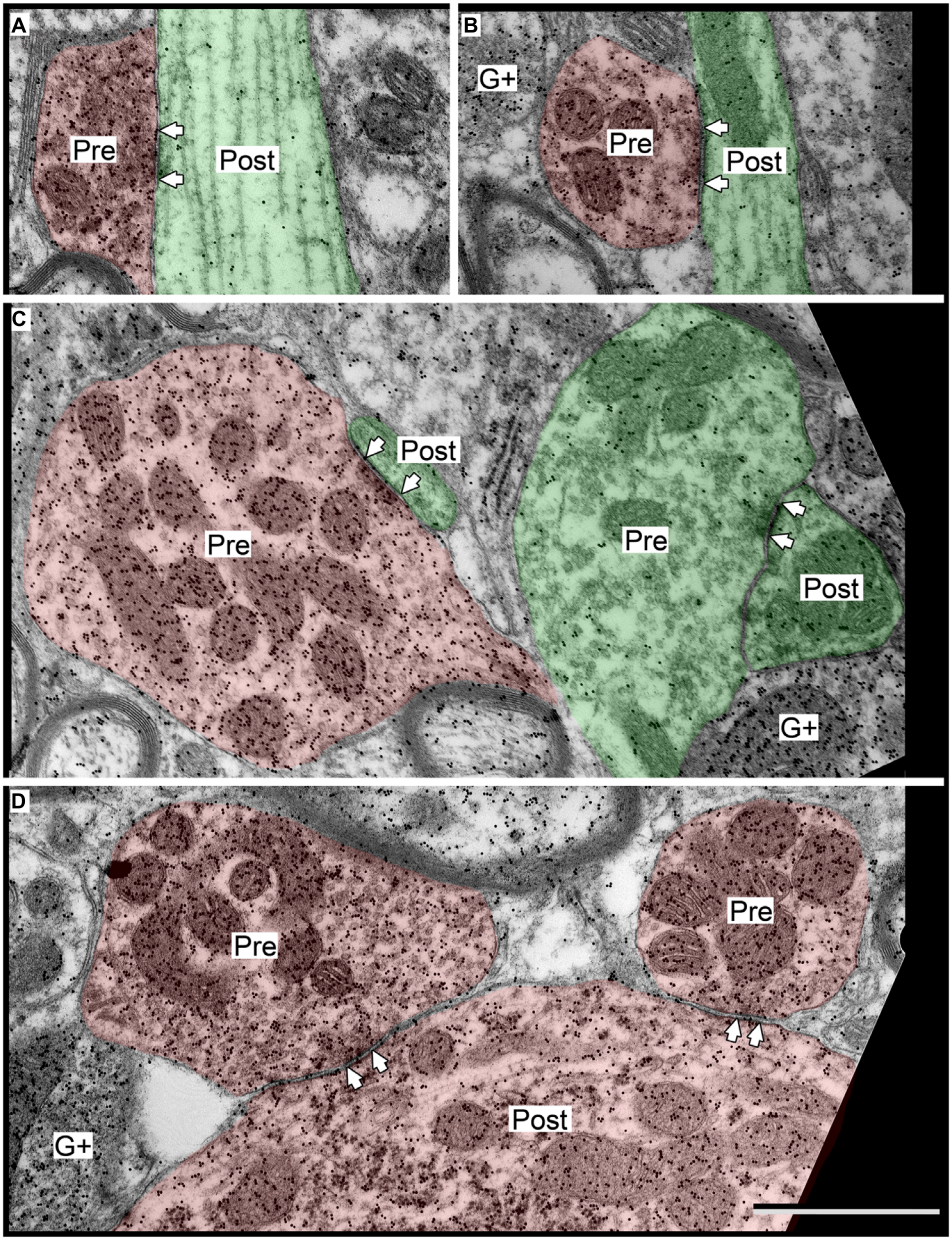
**Electron micrographs showing examples of GABAergic synapses in the ICc. (A,B)** SG boutons contacting excitatory shafts. **(C)** LG bouton contacting an excitatory spine. A synapse with an excitatory bouton (green “Pre”) contacting an excitatory shaft is also visible. **(D)** Two LGs contacting a GABAergic soma. Scale bar = 1 μm. Conventions as in **Figure 2**.

**FIGURE 9:**
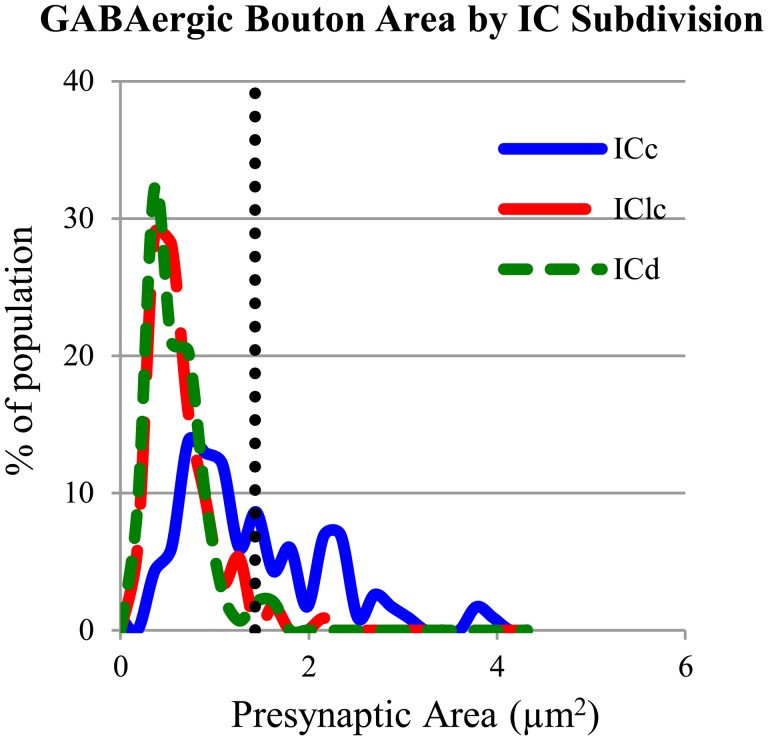
**Plot of the GABAergic presynaptic profile areas in ICc (solid blue line), IClc (dashed red line), and ICd (dashed green line).** The vertical dotted line indicates 3 SD above the mean of the boutons sizes in ICd and IClc. Only 2.1% of the ICd boutons and 2.7% of the IClc boutons occur above the line. A large proportion (36.8%) of boutons in ICc are larger than the majority of boutons in ICd or IClc. Sample sizes = 116 from the ICc; 114 from the IClc; 143 from the ICd.

The GABAergic presynaptic boutons in the ICc included small profiles similar to those in the IClc and ICd (**Figures 8A,B**), as well as much larger profiles (**Figures 8C,D**). The larger profiles also contained more mitochondria (compare the boutons in **Figures 8C,D** with the boutons in **Figures 4, 6, and 8A,B**). The apparent size difference was confirmed by quantitative analysis of profile areas (**Figure 9**). The distribution of bouton areas in the ICc (**Figure 9**, solid blue line) extended to much higher values than the areas of boutons in the IClc and ICd (**Figure 9**, green dashed line and red dashed line). We distinguished the larger GABAergic boutons in the ICc by establishing a threshold of three SD above the mean of the GABAergic bouton area in the IClc and ICd (**Figure 9**, dotted vertical line). GABAergic ICc boutons above this threshold (1.43 μm^2^) were classified as LG boutons (36.8% of the ICc boutons) and GABAergic ICc boutons below the threshold were classified as SG boutons (63.2% of the ICc boutons).

The LG and SG boutons differed in their targets. While both types targeted excitatory cells more often than GABAergic cells (83% of LG targets and 89% of SG targets), there were differences in the parts of IC cells that each bouton type contacted (**Table 2**). SG boutons contact excitatory dendritic shafts most often, but also contact excitatory spines, excitatory somas, and GABAergic somas. A smaller percentage of LG synapses make contacts on excitatory shafts, and larger percentage make contacts on excitatory spines, excitatory somas, and GABAergic somas.

**Table 2:** Distributions of the post-synaptic targets of the two classes of GABAergic boutons in the ICc.

ICc	Excitatory targets				GABAergic targets				
	Soma (%)	Shaft (%)	Spine	Exc. subtotal (%)	Soma (%)	Shaft (%)	Spine (%)	GABA subtotal (%)	Total (%)
Small GABAergic boutons	5	67	17	**89**	7	4	0	**11**	100
Large GABAergic boutons	17	37	29	**83**	12	5	0	**17**	100

*Each row totals 100%. Exc., excitatory.*

### GABAergic AND EXCITATORY SYNAPSES HAVE DIFFERENT DISTRIBUTIONS ON ICc CELLS

In the ICc, comparisons of GABAergic synapses with our previous data on excitatory synapses also suggested that these boutons are distributed differently with respect to postsynaptic profile identity. As in the other subdivisions, both GABAergic and excitatory synapses form with excitatory targets much more often than with GABAergic targets (**Table 1**, ICc).

**FIGURE 10:**
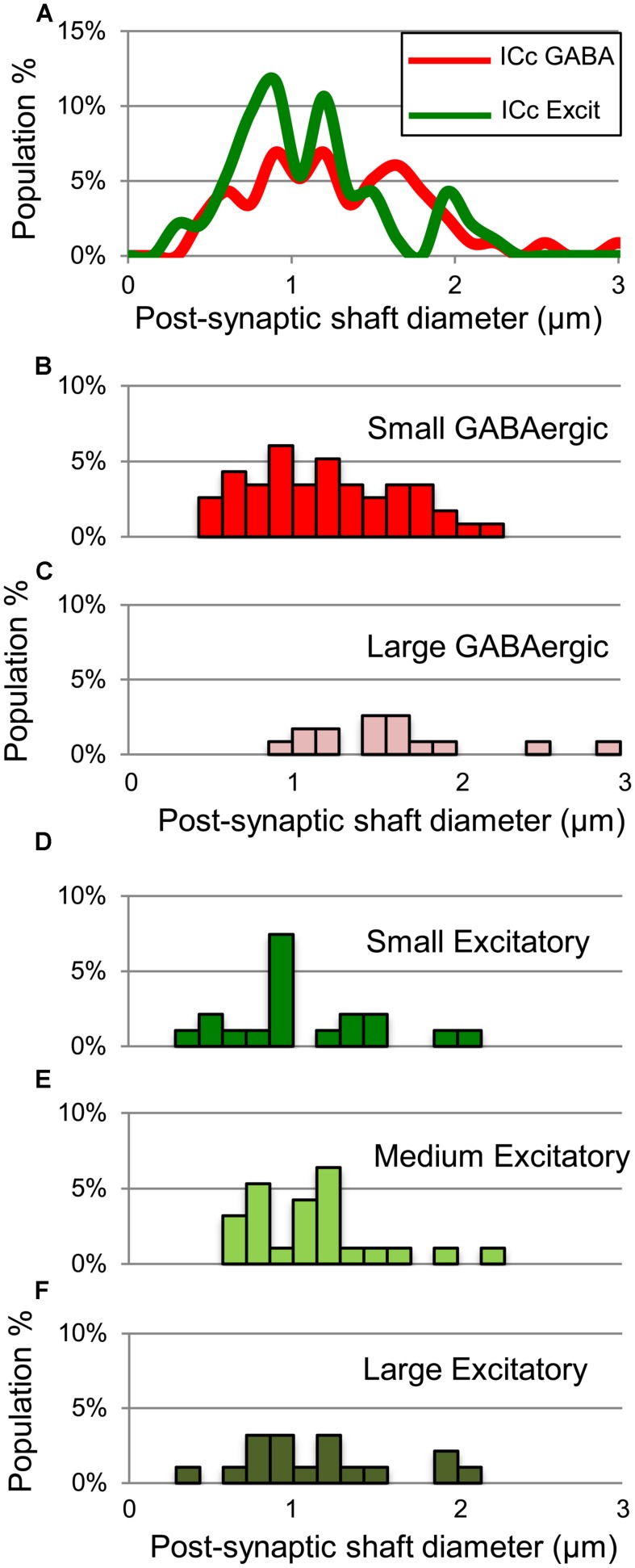
**Plots showing the diameters of excitatory dendritic shafts that are part of GABAergic synapses (red) or excitatory synapses (green) in the ICc. (A)** Line graphs comparing the diameters of target dendrites for all GABAergic synapses (red) vs. all excitatory synapses (green). **(B,C)** Histograms showing the diameters of the post-synaptic dendritic shafts for Small or Large subtypes of GABAergic synapses. The y-axis is the population percentage for all GABAergic synapses in the ICc. **(D–F)** Histograms showing the diameters of the post-synaptic dendritic shafts for Small, Medium or Large subtypes of excitatory synapses. The y-axis is the population percentage for all excitatory synapses in the ICc. Excit – excitatory.

For excitatory targets, the greatest difference between GABAergic and excitatory synapses was in the proportions of each that contact excitatory somas. These somas receive 10% of the GABAergic boutons but only 1% of the excitatory boutons (**Table 1**, ICc, excitatory targets). Both GABAergic synapses and excitatory synapses formed on excitatory spines (21% vs. 16%) and excitatory shafts (56% vs. 62%). We did not find significant differences in the size of the postsynaptic excitatory shafts between the GABAergic synapses and the excitatory synapses (**Figure 10A**). Excitatory synapses in the ICc include a class, the LE bouton, that rarely occur in the other IC subdivisions (Nakamoto et al., 2013a). We did not find an obvious difference in the size of the postsynaptic excitatory shafts on the GABAergic and excitatory synapse subtypes (**Figures 10B–F**).

Contacts onto GABAergic neurons also revealed differences between excitatory and GABAergic inputs (**Table 1**, ICc, GABAergic Targets). Both GABAergic and excitatory boutons contacted the shafts and somas of GABAergic targets. However, excitatory boutons targeted GABAergic shafts more frequently than GABAergic boutons.

## DISCUSSION

The present study describes the ultrastructure of GABAergic synapses in the IC and compares them to excitatory synapses. We identified differences in the postsynaptic targets of GABAergic and excitatory synapses. In the IClc and ICd, GABAergic synapses more frequently form on excitatory somas and, on average, target larger dendritic shafts, while excitatory synapses more frequently form on excitatory spines and, on average, target smaller dendritic shafts. These differences in the postsynaptic targets of the GABAergic and excitatory synapses in the IClc and ICd suggest functional implications. The synaptic organization in the IClc and ICd differs substantially from the organization in ICc. In the ICc, we identified two populations of GABAergic boutons: LG boutons and SG boutons. The LG boutons are reminiscent of lemniscal GABAergic boutons (Oliver and Shneiderman, 1989; Oliver et al., 1995). We will discuss the relationship of this report to previous studies of GABAergic synapses in the IC and then discuss some implications of the synaptic organization.

### TECHNICAL CONSIDERATIONS

We used a GABA antibody that has been used in numerous brain areas and numerous species, including guinea pigs, rats, rabbits, cats, and mice (Coomes et al., 2002; Ruiz et al., 2004; Ponti et al., 2008; Luzzati et al., 2009; Brown et al., 2012; Smith et al., 2012). The GABA-positive profiles in the present study (boutons, somas, dendrites, and spines) have ultrastructural features that are similar to previous descriptions of GABAergic structures in the IC (Roberts and Ribak, 1987; Oliver and Beckius, 1992). With any antibody, there is a concern about over- or under-staining. We very rarely observed GABA-positive boutons with excitatory ultrastructural features, so we do not believe there was significant overstaining. Understaining can be harder to assess, but we observed GABA-positive spines, which are among the more difficult profiles to stain with anti-GABA antibodies; consequently we do not think that substantial understaining occurred.

In this report we described the distribution of GABAergic and excitatory synapses in three IC subdivisions, several caveats apply. First, for each subdivision, we distinguished targets only as GABAergic or non-GABAergic. While this distinction includes all IC cells, it fails to discriminate additional subtypes of cells (Morest and Oliver, 1984; Oliver et al., 1991; Malmierca et al., 1993, 1995; Sivaramakrishnan and Oliver, 2001). Whether, and how, these types differ in their patterns of synaptic inputs remains to be determined. This is a critical point, but at present there are no ultrastructural features that would allow us to assign the various postsynaptic profiles to specific cell types. The present data suggest, as a starting point, that synapse distributions are different for GABAergic and excitatory cells in each of the subdivisions examined. Further studies will be required to relate the distributions to individual cell types in the IC and show the distribution of all synapses on individual cells.

The use of ultrathin sections, without three dimensional reconstruction of serial sections, carries inherent limitations. A section through one SG bouton will be small regardless of the location of the section through the bouton. On the other hand, a section through one LG bouton could range from small to large. We analyzed only those profiles that contained a clear synaptic zone, which should eliminate many of the smaller, “tangential” sections through a bouton. However, it remains possible (perhaps likely) that some profiles through large boutons could appear small. We cannot rule out inclusion of profiles that would be “deceptively” small in area; such an error would affect the calculated proportions of SG and LG boutons in the ICc. However, it seems unlikely that a sampling bias could occur that could completely explain the rarity (<3%) of large profiles in the ICd and IClc. The differential distribution, with LG boutons almost completely restricted to the ICc, is strong evidence for a separate LG class of GABAergic bouton.

### COMPARISON WITH PREVIOUS STUDIES OF IC GABAergic SYNAPSES

This is the first report to describe the structure and distribution of GABAergic synapses in the IC of the guinea pig, a species used commonly in auditory research. This report expands upon the previous studies of GABAergic synapses in the IC in two ways: first, this report identifies the targets of GABAergic boutons in all subdivisions of the IC. Second, this report demonstrates that the largest GABAergic boutons occur almost exclusively in the ICc. Previous electron microscopy (EM) studies have concentrated on the ICc and hence comparisons between reports must be limited to this subdivision. Our results show that GABAergic synapses in the ICc of the guinea pig are similar to inhibitory synapses in the ICc of the rat and cat (Rockel and Jones, 1973; Roberts and Ribak, 1987; Shneiderman and Oliver, 1989; Oliver and Beckius, 1992; Paloff and Usunoff, 1992), in that they have symmetric junctions and pleomorphic vesicles. Across the studies (Rockel and Jones, 1973; Roberts and Ribak, 1987; Paloff and Usunoff, 1992; current report) it was noted that inhibitory synapses in the ICc most often contacted dendrites, while contacts onto spines and somas occur less frequently. The two studies that quantified the frequency of contacts onto these targets reported similar results, suggesting similarities across species (cat: Oliver and Beckius, 1992; guinea pig: present study).

#### SG vs. LG boutons

We distinguished the LG boutons in the ICc by establishing a threshold of three SD above the mean of the GABAergic bouton area in the IClc and ICd. This is likely conservative for distinguishing two classes of GABAergic boutons. However, the almost complete restriction of LG boutons to the ICc, and the difference in the postsynaptic profiles of LG and SG boutons strongly argues for two classes of GABAergic boutons.

The morphological differences between the LG and SG boutons raise a question of functional differences. Individual LG boutons may be more effective than SG boutons at inhibiting their targets. Large boutons have been associated with larger postsynaptic effects, which can be due to larger synaptic zones or to the release of larger numbers of vesicles (Pierce and Lewin, 1994). While we did not assess the area of the synaptic zones, the association between presynaptic area and postsynaptic effect suggest that these differences would be worthwhile to examine with three-dimensional reconstruction. The overall effect, of course, will depend on how many LG or SG boutons converge on a specific cell, and on the temporal pattern of activation of these converging inputs and on the relative locations of the active inputs.

#### Sources of GABA inputs to the IC

GABAergic boutons could arise from a wide range of sources. The major extrinsic sources of GABAergic input to the IC are the nuclei of the lateral lemniscus and the periolivary nuclei (especially the superior paraolivary nucleus; Adams and Mugnaini, 1984; Shneiderman and Oliver, 1989; González-Hernández et al., 1996; Zhang et al., 1998). Additional GABAergic inputs arise from the contralateral IC (González-Hernández et al., 1996; Hernández et al., 2006; Nakamoto et al., 2013c). In addition, ICc cells typically have extensive local collaterals (Oliver et al., 1991), so another source of GABAergic inputs to ICc is likely intrinsic GABAergic cells. Together, these sources must account for both the LG and the SG boutons, but remarkably few data are available for a detailed account. Shneiderman and Oliver (1989) described boutons in the IC that originated from the dorsal nucleus of the lateral lemniscus. These boutons are almost certainly GABAergic (Shneiderman et al., 1988). The study was done in cats, so possible species differences must be considered, but the boutons were similar to the LG class in the present study. The dorsal nucleus of the lateral lemniscus projects mainly to the ICc, consistent with the distribution of LG boutons. Further association of GABAergic sources with bouton type will require ultrastructural examination of selectively labeled inputs.

#### Distribution of synaptic inputs on IC cells

In general, the majority of inputs to excitatory IC cells are on the dendritic shafts, followed by spines, and finally the soma. Spines are present on many IC cells and are postsynaptic to both excitatory and inhibitory boutons. We noted three features that suggested that excitatory inputs have a more restricted effect than GABAergic inputs on excitatory cells in the ICd and IClc. First, in these subdivisions excitatory inputs frequently contacted spines and spines can compartmentalize biochemical signals. Second, for excitatory cells with tapering dendrites, the prominence of GABAergic inputs on larger dendritic shafts than excitatory inputs may allow for a more general inhibitory gating of distal excitatory inputs. Finally, we often noted GABAergic inputs, but not excitatory inputs, occurring on dendritic shafts as they arise from the soma. This arrangement of GABAergic and excitatory inputs is common in many brain areas and provides opportunities for selective actions of inhibitory and excitatory inputs (Megias et al., 2001; Kameda et al., 2012). This is particularly interesting in the ICd and IClc, which receive descending excitatory inputs from the auditory cortex (Nakamoto et al., 2013b). Of course, how two inputs can interact depends on whether they converge on the same structures. It is also possible that the difference in the targets of GABAergic and excitatory boutons may be due to a difference in target excitatory cells. GABAergic boutons may target excitatory cells with larger dendritic shafts and fewer spines, while the excitatory boutons may target excitatory cells with smaller dendritic shafts and more spines. Such information is beyond the scope of the present study but will be critical for the eventual understanding of synaptic integration in IC cells. The present study demonstrates that excitatory and GABAergic inputs are distributed differently (and that such differences extend to subtypes of excitatory and GABAergic synapses), and so future studies to examine interactions are likely to be fruitful.

In all IC subdivisions GABAergic cells receive GABAergic and excitatory input. Despite the present sample of over 300 synapses, the number on GABAergic cells was relatively small. This likely reflects the relative abundance of GABAergic vs. excitatory cells in the IC (approximately 20–25%; Oliver et al., 1994; Merchán et al., 2005; Mellott et al., 2014). Nonetheless, the evidence confirms that IC GABAergic cells receive both excitatory and GABAergic inputs of a variety of subtypes and that these inputs may be distributed differentially on the somas and dendrites.

#### Comparisons between IC subdivisions

There are both similarities and differences between the IC subdivisions in the detailed patterns of synaptic inputs. An obvious difference is the substantial presence of larger boutons in the ICc than in the other subdivisions. This includes both excitatory and GABAergic boutons, as evidenced by the presence of LE and LG boutons in the ICc. Such differences may reflect the “lemniscal” functions of the ICc. A second difference concerns the relative distributions of excitatory and GABAergic synapses on the target cell. In the IClc and the ICd, GABAergic boutons are biased toward somas and large dendrites whereas the excitatory boutons are biased toward spines and small dendrites, potentially allowing for strong inhibitory gating of excitatory inputs. In the ICc, GABAergic and excitatory boutons are distributed similarly along IC cells. This arrangement may underlie more restricted interactions (e.g., within individual dendrites) between excitatory and GABAergic inputs in the ICc. Beyond this, the similarities in distributions of synaptic inputs appear to outweigh the differences between subdivisions, suggesting that the varied functions proposed for the IC subdivisions may relate more to the differences in inputs and outputs than in the mode of integration within the individual cells. More substantial differences may become apparent as information about individual cell types becomes available.

## Conflict of Interest Statement

The authors declare that the research was conducted in the absence of any commercial or financial relationships that could be construed as a potential conflict of interest.

## ABBREVIATIONS

bic: brachium of the inferior colliculus
D: dorsal
GABAnegative: gamma-aminobutyric acid immunonegative
G-: gamma-aminobutyric acid immunonegative
GABA-positive: gamma-aminobutyric acid immunopositive
G+: gamma-aminobutyric acid immunopositive
IC: inferior colliculus
ICc: central nucleus of the inferior colliculus
ICd: dorsal cortex of the inferior colliculus
IClc: lateral cortex of the inferior colliculus
LE: large excitatory bouton type
LG: large GABAergic bouton type
LSO: lateral superior olive
ME: medium excitatory bouton type
Mo5: motor trigeminal nucleus
NADPH-d: nicotinamide adenine dinucleotide phosphate diaphorase
PB: 0.lMphosphate buffer
Pn: pontine nuclei
R: rostral
SC: superior colliculus
scp: superior cerebellar peduncle
SE: small excitatory bouton type
SG: small GABAergic bouton type
SN: substantia nigra
VLL: ventral nucleus of the lateral lemniscus
VNTB: ventral nucleus of the trapezoid body.
